# Biofilm formation of *Klebsiella pneumoniae* on urethral catheters requires either type 1 or type 3 fimbriae

**DOI:** 10.1111/j.1574-695X.2012.00965.x

**Published:** 2012-04-23

**Authors:** Steen G Stahlhut, Carsten Struve, Karen A Krogfelt, Andreas Reisner

**Affiliations:** 1 Department of Microbiological Surveillance and Research Statens Serum InstitutCopenhagen, Denmark; 2 Biomedical Science, University of Applied SciencesGraz, Austria

**Keywords:** CAUTI, type 1 fimbriae, biofilm, *Klebsiella pneumoniae*, type 3 fimbriae

## Abstract

Urinary catheters are standard medical devices utilized in both hospital and nursing home settings, but are associated with a high frequency of catheter-associated urinary tract infections (CAUTI). In particular, biofilm formation on the catheter surface by uropathogens such as *Klebsiella pneumoniae* causes severe problems. Here we demonstrate that type 1 and type 3 fimbriae expressed by *K. pneumoniae* enhance biofilm formation on urinary catheters in a catheterized bladder model that mirrors the physico-chemical conditions present in catheterized patients. Furthermore, we show that both fimbrial types are able to functionally compensate for each other during biofilm formation on urinary catheters. *In situ* monitoring of fimbrial expression revealed that neither of the two fimbrial types is expressed when cells are grown planktonically. Interestingly, during biofilm formation on catheters, both fimbrial types are expressed, suggesting that they are both important in promoting biofilm formation on catheters. Additionally, transformed into and expressed by a nonfimbriated *Escherichia coli* strain, both fimbrial types significantly increased biofilm formation on catheters compared with the wild-type *E. coli* strain. The widespread occurrence of the two fimbrial types in different species of pathogenic bacteria stresses the need for further assessment of their role during urinary tract infections.

## Introduction

Urinary catheters are standard medical devices utilized in both hospital and healthcare facilities. The most frequent complication associated with these devices is the development of nosocomial catheter-associated urinary tract infections (CAUTIs). Up to 50% of patients will at some point during their hospitalization be catheterized, severely increasing the patient's risk of colonization by microorganisms, which occurs in 5–10% of the patient's at each day of catheterization (Jain *et al*., 1995; Saint, 2000; Warren, 2001). *Klebsiella pneumoniae*, an important opportunistic pathogen frequently causing UTIs, septicemia, or pneumonia in immune-compromised individuals, is responsible for up to 10% of all nosocomial bacterial infections (Jarvis *et al*., 1985; Spencer, 1996; Podschun & Ullmann, 1998). *Klebsiella pneumoniae* is a frequent cause of CAUTIs (Nicolle, 2005; Macleod & Stickler, 2007; Ong *et al*., 2010; Wang *et al*., 2010). The severe complications of these infections caused by this bacterium are because of its intrinsic resistance to ampicillin but also its increasingly frequent multi-drug resistance.

Presence of indwelling urinary catheters favors biofilm formation of uropathogens such as *K. pneumoniae* and *Escherichia coli* by providing an inert surface for the attachment of bacterial adhesins, thereby enhancing microbial colonization and the development of biofilm (Macleod & Stickler, 2007; Jacobsen *et al*., 2008; Wang *et al*., 2010). Although the exact contribution of the catheter biofilm to pathogenicity is often unknown, the common recurrence of CAUTI soon after the completion of a course of antibiotic treatment is thought to be caused by re-colonization of the urine by organisms that have survived in the catheter biofilm. Thus, development of better catheter surfaces and improved treatment options requires a better understanding of the catheter attachment mechanisms.

Based on *in vitro* studies in various biofilm model systems, the attachment of bacteria to catheters is initiated by adhesins, for example, fimbriae, located on the bacterial surface (Jacobsen *et al*., 2008). The best understood fimbrial types of *K. pneumoniae* that are also the most frequently encoded fimbriae are type 1 fimbriae and type 3 fimbriae. Type 1 fimbriae are encoded by the majority of *Enterobactericeae* (Duguid *et al*., 1955). They belong to the chaperone–usher class fimbriae family and are encoded by the *fimABCDEFGHIK* gene cluster, with *fimA* being the major structural subunit while *fimH* encodes for the adhesive subunit, FimH, which was shown to mediate adhesion to mannose-containing structures present on host tissue surfaces and extracellular matrix (Klemm *et al*., 1990; Krogfelt *et al*., 1990; Madison *et al*., 1994). Type 1 fimbrial expression is phase variable, that is, mediated by an invertible DNA element (*fim* switch) (Klemm & Schembri, 2000; Struve *et al*., 2008). We recently established that type 1 fimbriae are essential for the ability of *K. pneumoniae* to cause urinary tract infections (Struve *et al*., 2008). Like type 1 fimbriae, type 3 fimbriae belong to the chaperone–usher class fimbriae. Apart from *Klebsiella* spp., type 3 fimbriae are commonly found in *Citrobacter*, *Enterobacter*, *Serratia*, *Proteus*, and *Providencia* isolates (Adegbola & Old, 1982, 1983, 1985; Old & Adegbola, 1982, 1983; Mobley & Chippendale, 1990). Recently, two independent studies reported plasmid-encoded type 3 fimbriae in independent *E*. *coli* strains (Burmølle *et al*., 2008; Ong *et al*., 2008). The fimbriae are encoded by the *mrkABCDF* gene cluster. The *mrkA* gene encodes the major structural subunit, while the *mrkD* gene encodes the fimbrial adhesin (Allen *et al*., 1991; Langstraat *et al*., 2001; Jagnow & Clegg, 2003). *In vitro* studies have shown that type 3 fimbriae mediate adhesion to different structures in human kidney and lung tissue and epithelial cells from human urine sediments, as well as to endothelial and bladder epithelial cell lines. However, the identity of the receptor remains elusive (Tarkkanen *et al*., 1990, 1992, 1997; Hornick *et al*., 1992). Furthermore, type 3 fimbriae were established to play an essential role in *K. pneumoniae* biofilm formation (Langstraat *et al*., 2001; Di Martino *et al*., 2003; Jagnow & Clegg, 2003; Schroll *et al*., 2010). Notably, both studies that identified plasmid-encoded type 3 fimbriae in *E*. *coli* reported that type 3 fimbriae expression profoundly enhanced the ability of *E*. *coli* to form biofilms *in vitro* (Burmolle *et al*., 2008; Ong *et al*., 2008).

Although both fimbrial types are well described in the literature, the exact contribution of *K. pneumoniae* type 1 and type 3 fimbriae on colonization and biofilm formation on urinary catheters is unknown. As culturing conditions have a dominant influence on average gene expression in biofilms and mutant analysis has also revealed that the requirement for several adhesins in efficient biofilm formation is not universal, but instead manifest only under certain conditions, it is important to evaluate the contribution of the *K. pneumoniae* adhesins to CAUTI pathogenicity under conditions that mirror best the situation in the human host (Beloin & Ghigo, 2005; Van Houdt & Michiels, 2005; An & Parsek, 2007).

In this study, we evaluated expression and the role of *K. pneumoniae* type 1 and 3 fimbriae in biofilm formation on urinary tract silicone catheters during growth in human urine using a catheterized bladder model that mirrors the physico-chemical properties during human infection. Our results reveal that expression of at least one of the fimbrial types for *K. pneumoniae* is required for efficient catheter colonization.

## Materials and methods

### Human urine as growth media

All experiments were performed in pooled human urine. For each experiment, human urine was collected from 4 to 10 healthy volunteers (both men and women) who had no history of UTI prior to collection. The urine was then pooled, filter sterilized through a filter cartridge (SARTOBRAN P, 0.2 μm; Sartorius, UK), and kept at 4 °C until use within the following 2 days.

### Bacterial strains and plasmids

Bacterial strains and plasmids used in this study are listed in Table 1. Bacteria were routinely cultured at 37 °C in Luria–Bertani (LB) broth supplemented with 100 μg mL^−1^ ampicillin when appropriate or in pooled human urine.

**Table 1:** Bacterial strains and plasmids used in this study

Strains and plasmids	Description	Reference
Strains		
C3091	*K. pneumoniae* UTI isolate	Oelschlaeger & Tall (1997)
C3091Δ*fim*	Type 1 fimbriae cluster deleted in C3091	Struve *et al*. (2008)
C3091Δ*mrk*	Type 3 fimbriae cluster deleted in C3091	Struve *et al*. (2009)
C3091Δ*mrk*Δ*fim*	Type 1 and 3 fimbriae cluster deleted in C3091	Struve *et al*. (2009)
HB101	Nonfimbriated, noncapsulated *E. coli* K-12 strain	Purcell and Clegg (1983)
Plasmids		
pUC18	Plasmid vector, *bla*	Yanisch-Perron *et al*. (1985)
pCAS625	pUC18 containing the cloned type 3 fimbriae gene cluster from C3091	Struve *et al*. (2009)
pCAS624	pUC18 containing the cloned type 1 fimbriae gene cluster from C3091	Struve *et al*. (2008)
Fosmids		
pEpi-FOS-5	Fosmid vector pEpi-FOS-5	Stahlhut *et al*. (2010)
pEpiFosfim	pEpi-FOS-5 containing the cloned type 1 fimbriae gene cluster from C3091	Struve *et al*. (2008)
pEpiFosmrk	pEpi-FOS-5 containing the cloned type 3 fimbriae gene cluster from C3091	Schroll *et al*. (2010)

### Preparation of type 3 fimbriae antiserum

Type 3 fimbriae were purified as described previously (Tchesnokova *et al*., 2008) with a few modifications. Briefly, an *E*. *coli* strain expressing type 3 fimbriae encoded by plasmid pCAS625 was grown with shaking overnight in LB broth at 37 °C. The cells were harvested, resuspended in buffer (5 mM Tris, 15 mM NaCl, pH 7.4), and agitated in an osterizing blender. Cell debris was removed by centrifugation. Fimbriae were repeatedly precipitated by ammonium sulfate [(NH_4_)_2_SO_4_] to a final concentration of 40% (NH_4_)_2_SO_4_ and dialyzed against 5 mM Tris, 15 mM NaCl, pH 7.4 buffer. Finally, the fimbriae were resuspended in 50 mM Tris, 150 mM NaCl, and pH 7.4 and separated on sodium dodecyl sulfate polyacrylamide gels. The protein concentration was measured using a bicinchoninic acid protein assay kit (Pierce). The purification of type 3 fimbriae was verified by mass spectrometry. Polyclonal antibodies raised in chickens against purified type 3 fimbriae were obtained from Covalab (UK).

### Catheterized bladder model

The catheterized bladder model was performed as previously described [Stickler *et al*., 1999, (also containing a schematic illustration of the model)] with a minor modification. In brief, two-compartment (inner and outer compartment) glass chambers were maintained at 37 °C by circulation of water through the outer compartment. All components of the model (tubings, glass chambers, and adaptors) were sterilized by autoclaving. A size 14 sterile Foley all-silicone catheter (Bard, Denmark) was inserted into the inner compartment of the glass chambers through an outlet at the base, followed by inflation of the catheter retention balloon with 10 mL sterile water. In contrast to the original model used by Stickler and coworkers, the outer glass compartment is 5 cm longer than the inner compartment, assuring temperature maintenance of the top 10 cm of the catheter. The catheters were connected to standard drainage bags. Sterile urine (15 mL) was pumped (Watson–Marlow 205S) into the inner chambers to a level just below the eye holes of the catheter tips.

For inoculation, 0.5 mL of bacterial overnight cultures grown in LB at 37 °C and 250 r.p.m. for 16 h was transferred to 20 mL of sterile pooled human urine (37 °C) and incubated for 3 h at 37 °C and 250 r.p.m. For inoculation of the catheterized bladder models, an appropriate aliquot of the preculture representing 5 mL of an OD_600 nm_ of 0.2 (˜ 0.5 × 10^8^ CFU) was added to the urine in the inner chambers. After 1 h, the urine supply was resumed for 70 h at a constant rate of 30 mL h^−1^. For quantification of biofilm on catheters, the urine flow was stopped, and a sample of the bladder (inner compartment) content was collected. The inserted catheters were carefully removed from the bladder and cut aseptically. The tip of the catheter (including the eyeholes) was transferred to 1 mL saline (0.9% NaCl) solution. Following a rinse of the remaining catheter with 2 mL of saline to remove traces of bladder content, the retention balloon section was removed. The next 2-cm catheter section was transferred to 2 mL saline solution and served as catheter sample.

The bladder sample as well as the tip of the catheter and catheter samples, respectively, were sonicated for 5 min (50% Power) followed by vortexing for additional 2 min. Then the samples were serial diluted and plated on appropriate agar plates. Based on colony counts of the plates, CFU mL^−1^ bladder content, CFU cm^−1^ catheter, or CFU cm^−2^ catheter were calculated. The experiment was repeated three times independently using different batches of urine.

### Biofilm formation on catheters under hydrodynamic conditions

Biofilm formation under hydrodynamic conditions on sterile Foley all-silicone catheters (Bard) was carried out using a system previously described (Christensen *et al*., 1999; Ferrieres *et al*., 2007), with some modification. Briefly, 4.5-cm-long catheter pieces were connected to a Watson–Marlow 205S pump using silicone tubings. The system was sterilized with 0.5% sodium hypochlorite for 3–4 h and washed extensively with sterile water, prior to connection of the sterile catheter pieces. After connection of the catheter, the system was filled with prewarmed (37 °C) urine, and the flow was left on for 2 h to precondition the catheter surface. Then, overnight cultures standardized to OD_600 nm_ of 0.05 were inoculated in each channel, and bacteria were established in the catheters for 1 h before the flow was turned on at a rate of 1 mL h^−1^ and the system placed at 37 °C for 48 h. Hereafter, the catheter pieces were disconnected from the system and connected to a 1-mL syringe. Cells attached to the surface of the catheter were stained by aspiring 0.1% crystal violet (CV) through the catheter by the syringe and left for 20 min. Catheters were then washed three times in phosphate-buffered saline (PBS) to remove excess CV and subsequently dried. Finally, CV was dissolved in 1 mL of 96% ethanol and measured at A_595 nm_. A catheter piece treated the same way without any contact to bacterial cells served as blank. The experiment was repeated three times using different batches of urine.

### Immunofluorescence microscopy

Bacteria scraped from catheters and resuspended in 1 mL of PBS, or grown in suspension in pooled human urine for 70 h at 37 °C, were harvested by centrifugation at 4000 ***g*** and then fixated in 100 μL of 4% formalin in PBS. The cells were fixated for 20 min at 4 °C, followed by washing with PBS. Appropriate numbers of fixated bacteria were added to wells of a poly-l-lysine-coated six-well glass slides (Novakemi Ab) and air dried. Slides were washed in PBS for 10 min. Primary polyclonal antibodies raised against purified type 1 fimbriae (Stahlhut *et al*., 2009) and type 3 fimbriae in rabbits and chicken, respectively, were diluted 1 : 1000 in PBS, mixed together, and added to each well on the glass slide. After incubation in a humid chamber for 45 min, the slide was washed three times in PBS and incubated with 1 : 500 dilutions of secondary antibodies (Alexa Fluor 594-conjugated goat anti rabbit IgG (Invitrogen) for detection of type 1 fimbriae and Alexa Flour 488-conjugated goat anti chicken IgY (Invitrogen) for detection of type 3 fimbriae). After incubation in a dark humid chamber for 45 min, the slide was washed three times in PBS, air dried, and a cover slip was mounted with Vectashield mounting medium (Vector Laboratories, Burlingame, CA). Finally, the slide was examined with epifluorescence microscopy using appropriate excitation filters. Visual counting of fluorescence signals on randomly chosen objective positions was used to assess the percentage of cells expressing type 1 or type 3 fimbriae.

### Fimbrial switch orientation assay

A modification of a previously described method was used to determine the orientation of the *K. pneumoniae fim* switch (Struve & Krogfelt, 1999; Struve *et al*., 2008). All culture and catheter samples were boiled for 5 min. in PBS immediately after collection and then kept at −20 °C until used. After thawing, the culture and urine samples were centrifuged at 12 000 ***g*** for 15 min, and the supernatant was used as template for PCR. Primers CAS168 (GGGACAGATACGCGTTTGAT) and CAS169 (GGCCTAACTGAACGGTTTGA) were used to amplify an 817-bp region containing the *fim* switch with the Expand high-fidelity PCR system (Roche). Finally, the PCR products were digested with HinfI and separated on a 1.2% agarose gel.

## Results

### Role of fimbriae during biofilm formation in the catheterized bladder model

To investigate the role of type 1 and type 3 fimbriae in biofilm formation on urinary catheters under conditions mimicking the conditions in the patients, we utilized an *in vitro* model of a catheterized bladder that mirrors all physico-chemical properties except for the presence of a bladder epithelium (Stickler *et al*., 1999). Using pooled human urine as growth medium, the *K. pneumoniae* urinary tract isolate C3091, its isogenic type 1 fimbriae mutant (C3091Δ*fim*), type 3 fimbriae mutant (C3091Δ*mrk*), and type 1 and type 3 fimbriae double mutant (C3091Δ*fim*Δ*mrk*) were inoculated in the artificial bladder lumen at an initial concentration of ˜ 0.5 × 10^8^ CFU mimicking an established CAUTI. After 70 h of urine supply at ˜ 630 mL day^−1^, bacterial counts were monitored in the bladder lumen, on the catheter tip (located in the bladder), and on the lower part of the catheter (below the retaining balloon of the catheter).

**Fig. 1:**
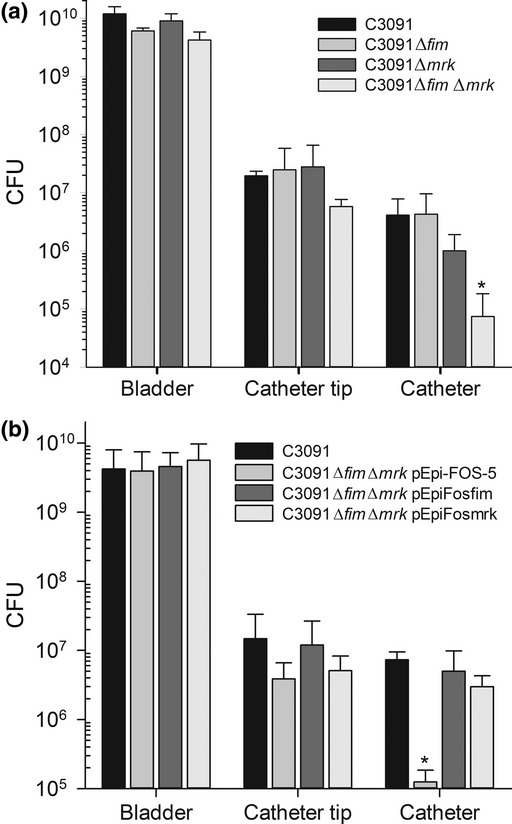
(a) Colonization of catheterized bladder model by wild type C3091 and C3091Δ*fim*, C3091Δ*mrk* and double mutant C3091Δ*fim*Δ*mrk*. (b) Colonization of catheterized bladder model by wild type C3091 and double mutant C3091Δ*fim*Δ*mrk* carrying different complementation constructs pEpi-Fos-5 (control), pEpiFosfim (type 1 fimbriae), and pEpiFosmrk (type 3 fimbriae). Each bar represents total CFU in the bladder or catheter tip and CFU per cm of catheter after 70 h of cultivation using pooled urine (M ± SE, *n* = 3). Statistical significant differences (*P* < 0.05) to wild type C3091 colonization are indicated by an asterisk.

All strains were found to grow to similar numbers in the bladder (Fig. 1), suggesting that no mutant was attenuated for survival in the bladder. In addition, this suggested that the number of bacteria transferred from the bladder into the catheter was similar for the wild-type and the mutant strains. Quantification of biofilm formation from the catheter tip showed that the wild-type and the type 1 fimbriae and type 3 fimbriae mutants formed similar amounts of biofilm. In contrast, the type 1 and type 3 double mutant showed a 3.4-fold attenuation in biofilm formation (Fig. 1a). On the catheter itself, for the wild-type and the type 1 fimbriae mutant, comparable numbers of cells were recovered from the catheter biofilm. However, for the type 3 fimbriae mutant a 4.1-fold attenuation in biofilm formation was indicated. Interestingly, for the type 1 and type 3 fimbriae double mutant, C3091Δ*fim*Δ*mrk* a significant 38-fold attenuation of biofilm formation compared with the wild-type strain (*P* < 0.05) was observed.

To confirm that at least one fimbrial type is sufficient for catheter colonization, biofilm formation in the catheterized bladder model of C3091Δ*fim*Δ*mrk* carrying expression cassettes for type 1 fimbriae and type 3 fimbriae in trans on stable fosmids EpiFosfim and EpiFosmrk, respectively, was evaluated. Indeed, compared with the control strain carrying the empty fosmid pEpi-FOS-5, colonization of catheter lumen by C3091Δ*fim*Δ*mrk* carrying pEpiFosfim and pEpiFosmrk was improved by 40 and 24-fold, respectively (Fig. 1b). Colonization of complemented strains was not significantly different to wild-type C3091 (*P* > 0.05). We concluded that absence of both fimbriae in *K. pneumoniae* leads to a significantly reduced catheter colonization and that complementation with either one fimbrial type restores catheter colonization to wild-type level.

### Type 1 fimbrial expression is up-regulated in cells colonizing urethral catheters

**Fig. 2:**
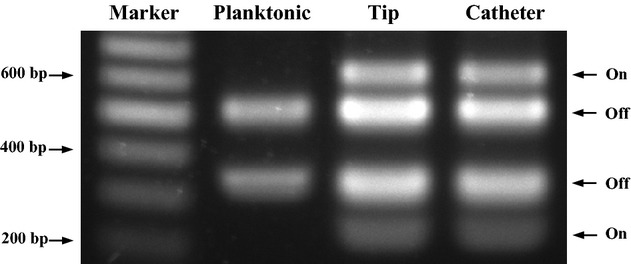
Orientation of the *fim* phase switch during planktonic growth and biofilm formation on urinary catheters by wild type, C3091. Lane 1, molecular size marker. Lane 2, planktonic growth. Lane 3, biofilm formation catheter tip (in the bladder). Lane 4, biofilm formation on catheter. ‘On’ denotes, that *fim* switch is transcribing *fimA*, while ‘off’ denotes no transcription of *fimA*.

Attenuation of catheter biofilm formation in the absence of type 1 fimbriae and type 3 fimbriae suggested that expression of both fimbrial types is important for catheter colonization or during biofilm formation. We, therefore, expected that expression of both fimbrial types would be detectable in bacterial samples from the colonized catheter. As expression of *K. pneumoniae* type 1 fimbriae is regulated at the transcriptional level by inversion of an invertible DNA element (*fim* switch), we measured the abundance of cells carrying the invertible element in the ‘on’ position within samples harvested from the catheter surface colonized with *K. pneumoniae* C3091. Interestingly the ‘on’ orientation was detected at high ratios both from bacteria forming biofilm on the catheter tip as well as in the catheter itself (Fig. 2, tip and catheter). In contrast, in the cells that were grown in suspension in urine for 70 h, only fragments corresponding to the ‘off’ orientation were detectable (Fig. 2). We concluded that transcription of the genes involved in type 1 fimbriae production are strongly induced in cells from catheters and that biofilm on urethral catheters are a mode of growth that leads to up-regulation of type 1 fimbriae expression in *K. pneumoniae*.

### Type 1 and type 3 fimbriae are expressed during biofilm formation on urinary tract catheters, but not in planktonic cells grown in urine

**Fig. 3:**
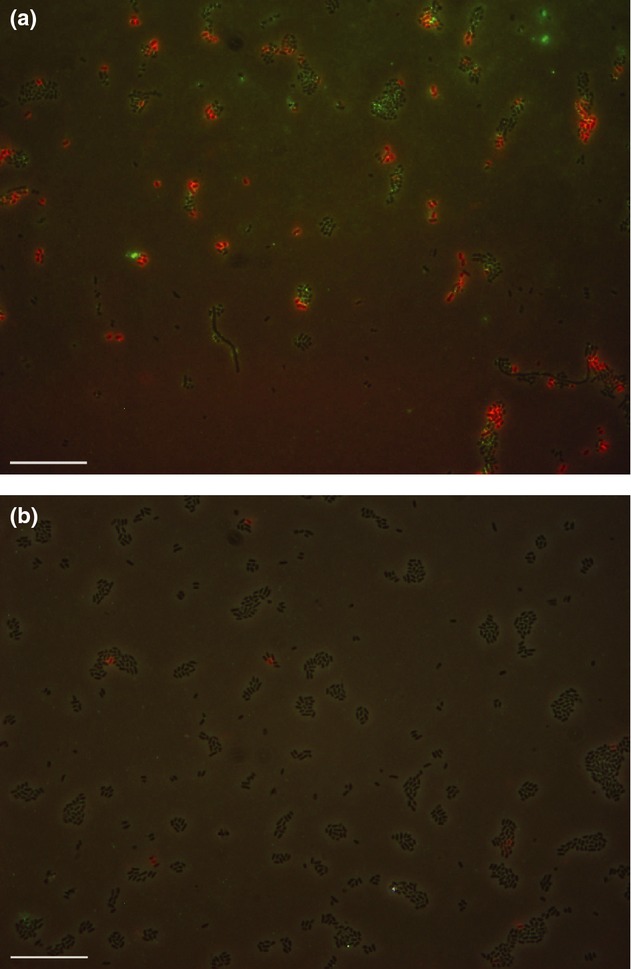
Expression of type 1 (Red) and type 3 (Green) fimbriae by individual *Klebsiella pneumoniae* C3091 cells grown as a biofilm on urinary tract catheters (a) or planktonically in urine (b) using type 1 fimbriae specific antibodies and type 3 fimbriae specific antibodies. Black cells are non-fimbriated cells. Note that during biofilm formation on urinary catheters each individual cell is expressing either type 1 or type 3 fimbriae not both. Scale bars denote 20 μm.

Onset of transcription of type 1 fimbriae upon colonization of catheters suggested increased fimbriae elaboration by colonizing cells. To investigate the expression of type 1 and type 3 fimbriae on the surface of biofilm-forming cells from catheters, immunostaining using polyclonal antibodies against type 1 and type 3 fimbriae, respectively, was performed. Prior to application on biofilm-forming cells, antibodies were tested for specificity toward type 1 and type 3 fimbriae, respectively. Both antibodies demonstrated high specificity toward the intended fimbriae, and no cross-reaction was observed when tested against C3091Δ*fim*, C3091Δ*mrk*, C3091Δ*fim*Δ*mrk* (data not shown). We then applied the identical immunostaining method on biofilm cells removed from the catheter section just below the retaining balloon following 70 h urine supply in a catheterized bladder model colonized with *K. pneumoniae* C3091 (Fig. 3a). Specific fluorescent signals indicating expression of either type 1 fimbriae or type 3 fimbriae (Fig. 3a) were observed in the vast majority of cells from catheter biofilms, revealing that both type 1 and type 3 fimbriae are indeed expressed during colonization and biofilm formation on urethral catheters. However, no cells expressing both fimbrial types simultaneously were observed (Fig. 3a). Thirty per cent of the cells were expressing type 1 fimbriae, and 60% of the cells were expressing type 3 fimbriae. In contrast, only very few cell expressed type 1, and no type 3 fimbriae-specific signals were detectable when cells were grown for 70 h in suspension indicating that type 1 and type 3 fimbriae are not expressed or only sparsely expressed under planktonic growth conditions (Fig. 3b). We concluded that urethral catheter colonization by *K. pneumoniae* is associated with expression of type 1 or type 3 fimbriae. However, fimbriae expression appears to be limited to only one type of fimbriae.

### Individual expression of type 1 and type 3 fimbriae in *E. coli* promotes biofilm formation on urethral catheters under hydrodynamic conditions

Our results suggested that type 1 and type 3 fimbriae expression is co-regulated in *K. pneumoniae*, limiting our ability to clarify the role of each fimbrial type for catheter colonization in *K. pneumoniae*. We, therefore, investigated the effect of type 1 and type 3 fimbriae expression for biofilm formation on urethral catheters in an otherwise nonfimbriated *E*. *coli* K-12 strain. *Escherichia coli* HB101 was transformed with type 1 (pCAS624) or type 3 fimbriae (pCAS625) encoding plasmids and investigated for biofilm formation under hydrodynamic conditions. Using pooled human urine as growth media, we utilized a system similar to the standard biofilm flow chamber system, but exchanged the flow chamber cells with pieces of silicone urethral catheters. After 48 h, biofilm formation on catheter pieces was quantified by crystal violet staining.

**Fig. 4:**
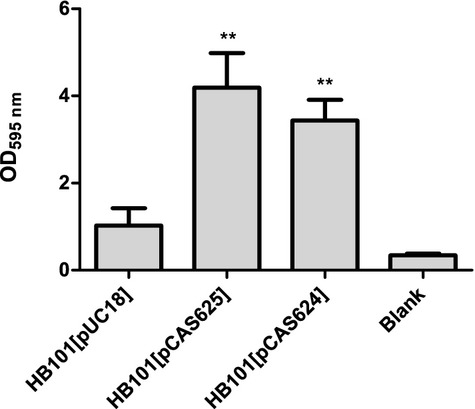
Biofilm formation on urinary tract catheters by the non-fimbriated *Escherichia coli* strain HB101[pUC18], HB101[pCAS624] expressing type 1 fimbriae and HB101[pCAS625] expressing type 3 fimbriae, quantified by crystal violet straining after 48 h. All experiments were performed in triplicates. Means are shown and statistical calculations done by Students *t*-test.

The introduction of pCAS624 encoding the type 1 fimbrial gene cluster of C3091 into *E. coli* HB101 resulted in significantly enhanced biofilm formation compared with HB101 carrying the empty vector pUC18 (*P* = 0.006) (Fig. 4), confirming that expression of type 1 fimbriae strongly promotes biofilm formation on urethral catheters. Similarly, a significantly enhanced biofilm formation was observed when pCAS625 encoding the type 3 fimbriae gene cluster of C3091 was transformed into HB101 (*P* = 0.007) (Fig. 4). These results suggest that *K. pneumoniae* fimbriae can be expressed in a nonfimbriated *E. coli* laboratory strain and that both fimbrial types can independently promote biofilm formation on urethral catheters.

## Discussion

Although animal models for CAUTI have been developed, the conditions present during human infection differ from current animal models in the urine composition as well as the residual level of urine in the human bladder and the continuous flow of urine through the catheter. In this study, we, therefore, assessed the contribution of genetic factors on catheter colonization in a dynamic *in vitro* model for the catheterized bladder.

We recently reported that type 3 fimbriae are essential for biofilm formation in a standard biofilm hydrodynamic flow chamber setup; however, type 1 fimbriae did not play any role in biofilm formation under the used experimental conditions (Schroll *et al*., 2010). In the present study, using a catheterized bladder model, we observed that the type 1 and type 3 fimbriae mutants were still able to form biofilms on catheters although the type 3 fimbriae mutant was attenuated. However, when both fimbrial types were deleted, a significant attenuation in biofilm formation was observed. Notably, the attenuation in biofilm formation between the wild-type and the type 1 and type 3 fimbrial double mutant is not significantly different on the catheter tip in contrast to below the retention balloon of the catheter. This suggests that the contribution of the fimbriae is particularly important for biofilm formation in the presence of increased shear forces caused by the flushing of the catheter lumen by the voiding urine.

These findings suggest that type 1 and type 3 fimbriae both promote biofilm formation on catheters and are able to compensate for each other. Thus, only when both fimbrial types are absent, the ability to form biofilm is severely attenuated. Based on this observation, we investigated the fimbrial expression in cells forming biofilm on urethral catheters by fimbriae-specific antibodies. Interestingly, both fimbrial types were expressed by C3091 during biofilm formation on the catheter surface, suggesting that none of the two fimbrial types are favored by the bacteria for promotion of biofilm formation. Furthermore, the inability to express both type 1 and type 3 fimbriae, when grown planktonically in urine as opposed to when forming a biofilm on catheters, suggests that the bacterial cell needs a substratum, for example, catheter or a biofilm, before transcription of these fimbrial types takes place. In addition, the high frequency of fimbrial expression in biofilm bacteria that were harvested after 70 h of biofilm formation provides evidence that *K. pneumoniae* requires either of the fimbrial types during biofilm maturation and not only during initial attachment.

Surprisingly, our analysis of fimbrial expression indicates that none of the individual cells expressed both type 1 or type 3 fimbriae simultaneously. This indicates a strict co-regulation system of the two fimbrial types in *K. pneumoniae*, where the expression of one fimbrial type will down-regulate the other fimbrial type. It could be speculated that several putative regulatory genes present in or up- and down-stream from the type 1 and type 3 fimbriae gene clusters might be part of a complex regulatory network. Thus, the *fimK* gene, which is unique to the *K. pneumoniae fim* gene cluster, has been shown to up-regulate type 1 fimbrial expression when deleted, and the *mrkH* gene has been shown to regulate type 3 fimbrial expression (Rosen *et al*., 2008; Johnson *et al*., 2011; Wilksch *et al*., 2011). Interestingly, both of these regulatory genes encode proteins that have internal domains capable of interaction with cycling di-GMP, a global bacterial second messenger believed to be involved in virulence regulation of many gram-negative bacterial species (Jonas *et al*., 2009; Struve *et al*., 2008; Bobrov *et al*., 2005; Johnson *et al*., 2011). Thus, c-di-GMP likely has a key role in the regulation of fimbrial expression in *K. pneumoniae*.

We recently reported that type 1 fimbriae was down-regulated (fimbrial switch is in the ‘off’ position) during biofilm formation in flow cells when grown in minimal media (Schroll *et al*., 2010). However, here we show that the type 1 fimbrial switch was ‘on’ during biofilm formation in urine on urethral catheters. Thus, the characteristics of substratum as well as the growth medium seem to play a key role in expression of type 1 fimbriae. The increased type 1 fimbriae expression may be related to the ability of the fimbriae to attach to the urethral catheter itself. However, type 1 fimbriae-mediated colonization and biofilm formation on catheters could also be enhanced by Tamm–Horsfall protein, which is found in abundance in human urine (Sokurenko *et al*., 1997; Cavallone *et al*., 2004). Type 1 fimbriae binds to Tamm-Horsfall protein, and we have previously shown that the binding groove of *K. pneumoniae* FimH adhesin has high tropism toward trisaccharides, which can be found on the surfaces of Tamm-Horsfall proteins. Thus, Tamm-Horsfall protein may coat the surface of the silicone catheter, thereby creating a layer of trisaccharides for the FimH adhesin to bind (Pak *et al*., 2001; Cavallone *et al*., 2004; Stahlhut *et al*., 2009). To evaluate the relevance of the latter possibility, it would be interesting to see whether biofilm formation of *K. pneumoniae* C3091 or its type 3 fimbriae mutant is reduced in the presence of mannose in the human urine.

When expressed in *E. coli*, both type 1 and type 3 fimbriae significantly enhanced biofilm formation compared with the wild-type strain, verifying that type 1 and type 3 fimbriae are important in biofilm formation on urethral catheters. These results indicate that type 1 and type 3 fimbriae are capable of promoting biofilm formation not only on urethral catheters when being expressed by *K. pneumoniae* but also in other Enterobacteriaceae.

Both type 1 and type 3 fimbriae have previously been suggested to adhere to urethral catheters using various experimental setups and artificial media (Burmølle *et al*., 2008; Trautner *et al*., 2008; Stahlhut *et al*., 2010; Wang *et al*., 2010). In this study, we utilized a catheterized bladder model and human urine, which provide a more precise simulation of CAUTIs than any other *in vitro* system, to establish the impact of type 1 and type 3 fimbriae on biofilm formation and characterize fimbrial expression.

Besides *Klebsiella* species, type 1 and type 3 fimbriae are found in many bacterial species causing catheter-associated urethral infections including *Citrobacter*, *Enterobacter*, *Serratia*, *Proteus*, *Providencia*, and *E. coli*, and we speculate that these fimbriae are involved in biofilm formation on urethral tract catheters in a large number of these species. Thus, further studies are warranted to elucidate the general role of these versatile fimbriae in different species causing CAUTIs. Detailed characterization of this process may lead to therapeutic means with anti-adhesive characteristics.

## Statement

Re-use of this article is permitted in accordance with the Terms and Conditions set out at http://wileyonlinelibrary.com/onlineopen#OnlineOpen_Terms
